# Some Seasonable Hints

**Published:** 1888-01

**Authors:** 


### SOME SEASONABLE HINTS.

One of the most common faults of our northern people is wearing an excess of clothing. One has only to look about him to note ladies, even at this early season, wrapped in furs, plush cloaks and other thick coverings, while any number of men are to be seen with overcoats sufficiently heavy to protect them from the severest cold in winter. To thus early become accustomed to such clothing is, of course, unwise in the extreme, and many must eventually pay the penalty of the habit. "Leave off your winter clothes late in the spring and put them on early in the autumn," is a wise injunction, but it must not be literally rendered. One should not, of course, thus early assume throughout his heaviest clothing, but the change should be made gradually. To put on warm under flannels early in the autumn is a sensible rule for all to follow. Equally as good a rule is to wear the fall overcoat as late as one can and be comfortable. Rather than trust entirely to clothing to keep warm, we should depend much upon exercise, and if too heavy wraps or overcoats are worn, that cannot be taken in sufficent amount to maintain good health, or when indulged in, the body is liable to become over-heated, and a "cold" is generally the consequence. A very common error with men oftener than women, is to wear, even in the coldest weather, too heavy outer clothing. Men generally select thick beaver and similar overcoats; and not a few prefer to have them made in the form of "ulsters," and thereby senselessly burden themselves with much additional weight. Such a garment also seriously interferes with walking and the long skirts are but little protection against cold, except in riding; certainly not sufficient to compensate for the inconvenience. Two thicknesses of clothing are always warmer than one equal in thickness to, or even thicker than, the two combined. With this fact all are familiar, and if they would take advantage of it in constructing winter garments the benefit would be considerable. A light weight cloak or overcoat lined with several thicknesses of flannel will be amply warm enough except when one is riding long distances and exposed to a piercing wind ; then, of course, an extra wrap, a shawl or cape is needed. Some women wear skirts made of felt, or similar compressed and compact material. They are highly objectionable, by reason of great weight, and again, they are less protection against cold than lighter fabrics of more open texture. Instead of one or more thick, heavy skirts, several lighter ones should be worn, and they will be found not only far more comfortable, but free from the evils which generally follows the use of the heavy garment.

Not only should the clothing be as light as comfort will permit, but it should be large enough to allow every movement of the body to be made with ease. This rule applies alike to men and women. What is considered a well-fitting coat is very generally too "tight across the chest," and, therefore, it interferes with breathing, or, at least, prevents deep inflation of the lungs. Some people may not know that when exposed to severe cold a feeling of warmth is really created by repeatedly filling the lungs to their utmost in this manner: Throw the shoulders well back and hold the head well up, inflate the lungs slowly, the air entering entirely through the nose. When the lungs are completely filled, hold the breath for ten seconds or longer, and then expire it quickly through the mouth. After repeating this exercise while one is "chilly," a feeling of warmth will be felt over the entire body, and even to the feet and hands. It is important for all to practise this exercise many times each day, and especially when in the open air. If the habit ever becomes universal, then consumption and many other diseases will rarely if ever, be heard of. Not only while practising the "breathing exercise" must the clothing be loose over the chest, but beginners will do well to remember, in having their clothing fitted, to allow for the permanent expansion of the chest of one, two and even three inches, which will eventually follow.

Some impurities are thrown out of the system by the skin, as others are by the lungs, the bowels and the kidneys. It is absolutely essential to health that the emanations from the skin, should pass easily through the clothing. This "transpiration" may be interfered with by an excess of clothing, or by clothing of a very close texture. All who wear india rubber coats know how uncomfortable they become after they have been on a short time. On the accession of Leo X. to the papacy, there was a grand procession at Florence in his honor. A little girl was made to personate the golden age, by being coated, from head to foot, with gold leaf. Before the day was over, she died in convulsions, killed because "transpiration," or, in other words, because carbonic acid gas, and dead, worn out matter, which should have been thrown out by her skin, were shut up in her system by the metallic covering. Ordinary clothing will not, of course, prevent transpiration, but an excess will interfere with it; and where too much clothing is worn the same soon becomes foul, unless the outside air can freely mingle with the gases from the body and so dilute them. Some wear the thickest and heaviest undervests which they can buy, and such people are very generally the victims of frequent colds. Following the rule of light clothing they would be much safer from the dangers of exposure were they to wear two light undervests instead of one very thick and heavy. Some advocate that the inner undervest should be of cotton and the outer of woolen. They are as a rule, well sustained, still there are some people who are subject to rheumatism and certain other difficulties, especially of the kidneys, around."
who do well to always wear woolen next to the skin "the year

A word about cold feet, of which very many complain and such people almost invariably suffer from indigestion and other derangements. An abbreviation of one of the ancient laws of health is "head cool and feet warm." An observance of this is certainly one of the primary essentials. If a person wears woolen stockings, as all ought to do in the winter, the most common cause of cold feet is sweating of the same, and it is the consequence of wearing shoes which are too tightly closed around the ankles. While the walking permits them to do so, people suffering from the trouble in question should wear low shoes and top gaiters. In very inclement weather, artics, worn over cloth shoes, are the best for them; when their feet are so dressed, they can walk in snow for hours without feeling the cold in the slightest degree. It ought to be unnecessary to add that a person should never go to bed with cold feet, for sleeplessness is one of the common consequences. Those who can bear it safely should, before retiring, immerse their feet in cold water for a moment, and then rub them briskly with a coarse towel until they are warm. For those in whom circulation is deficient, a hot brick or flat-iron, wrapped in flannel, is often essential, and should always be placed in the bed when needed.

In every heated room a thermometer is needed and should be carefully watched. 70 degrees of heat is all that any person needs who is comparatively well, and very many invalids would be far better if they never subjected themselves to a higher temperature. If one remains long in an overheated room he is quite certain to take cold when he goes out, unless he walks briskly until he has become accustomed to the change. Theatres, churches, etc., in the first place, and personal neglect in the second place, are accountable for many colds, sore throats, bronchial inflammations, etc. Such places are quite certain to be overheated and poorly ventilated, and people, especially ladies, are disinclined to remove their wraps. On leaving they enter a car or carriage and reach home shivering with the cold.

We find in neck wraps a common cause of "colds." If during winter one was certain to wear much the same wrap at all times when in the open air, there would be less danger from it. But the chances are that on some occasions when it should be worn it will be left off, either purposely or forgotten, and a cold is the result. The silk handkerchiefs with which many men adorn themselves cause more sore throats than any other influence. When once put on, the wearers are wedded to them for the remainder of the winter. The coat collar is ample protection when turned up and buttoned closely, and a man is very foolish to use any other means for the purpose. To attempt to criticize ladies' apparel, or at least that part of it worn about the neck, is beyond us, and we will merely leave them to draw conclusions from the advice which we give the men. There is another rule for the latter to observe. Always wear throughout cold weather the same style of collar, and be sure and have your shirts "cut high in the neck." If you wear a "dickey" to-day and a "turn down" to-morrow you are in for a sore throat, or something of that sort, the next day. No less important is it to wear continuously the same style of necktie.

Physicians are frequently asked if they recommend "chest protectors." They certainly should be worn if they will add to the comfort of the wearer. If a person has his vest "cut high," wears a broad necktie, has on warm flannels, and is otherwise properly dressed for winter, he will scarcely need a "chest protector," unless he suffers from some bronchial or pulmonary affection; in which one should be worn. In our climate, the so-called catarrhal troubles are very common; we refer especially to catarrh of the nose and throat. People who suffer from them take cold very easily, and are "stuffed up" after every trifling exposure. Such people should, of course, follow all the rules of health; but there is one which may be emphasized to meet their needs. Every morning on rising they should sponge the face, neck and uppermost part, at least, of the chest with water as cold as it runs from the faucet. That habit, if kept up, will not only save them many a cold, but will really help much in the treatment of their catarrh.

We must "toughen" ourselves to endure cold. We should depend more on exercise and less on clothing to keep our bodies warm. The clothing should be the lightest compatible with comfort. Cold sponge bathing or the air bath, is essential to the health of all who can properly employ either. We as a rule, need less heat in our houses and better ventilation. Personal care and the application of a little common sense "fill out the measure."

